# Supplementary material for: Patient hesitancy in perioperative clinical trial enrollment during the COVID-19 pandemic
Source: PLoS One. 2023 Jan 17;18(1):e0279643. doi: 10.1371/journal.pone.0279643 (PMC9844839; doi:10.1371/journal.pone.0279643)
Supplement: S1 Table — (DOCX) [file pone.0279643.s001.docx]

**S1 Table. NO/AKI Trial Patient Survey Questions and Response Types.**

| **Survey Question** | | |  | **Response Type** |
| --- | --- | --- | --- | --- |
| ***Informed Consent*** | | |  |  |
| **(1)** I understood how the study was going to be performed before participating. | | |  | Five-point Likert scale (strongly disagree to strongly agree) |
| **(2)** I was informed about the benefits of the study. | | |  | Five-point Likert scale (strongly disagree to strongly agree) |
| **(3)** I was informed about the risks of the study. | | |  | Five-point Likert scale (strongly disagree to strongly agree) |
| **(4)** The consent process was done in a professional manner. | | |  | Five-point Likert scale (strongly disagree to strongly agree) |
| **(5)** The researchers thoroughly answered all of my questions. | | |  | Five-point Likert scale (strongly disagree to strongly agree) |
| **(6)** The researchers were accessible to me when I needed to contact them. | | |  | Five-point Likert scale (strongly disagree to strongly agree) |
| **(7)** Was the study what you expected it to be? | | |  | Yes or no |
|  | If it was not what you expected, how was it different? Please explain below. Add any other comments pertinent to the consent process that you would like to share. | |  | Free response |
| ***Healthcare Experience*** | | |  |  |
| **(8)** Did this study make you more knowledgeable about the procedure you received? | | |  | Yes or no |
|  | | If you answered “yes” to the above question, please tell us what you learned below. |  | Free response |
| **(9)** Did participating in this study make you a more active part of the medical care you received? | | |  | Yes or no |
|  | | If yes, in what ways? |  | Free response |
| **(10)** How did participating in the study affect your level of stress or anxiety surrounding your procedure? | | |  | Five-point Likert scale (made me a lot more anxious to made me a lot less anxious) |
| ***Trial Satisfaction*** | | |  |  |
| **(11)** How satisfied or dissatisfied were you with your experience as a study participant? | | |  | Five-point Likert scale (very dissatisfied to very satisfied) |
| **(12)** Knowing what you know now, would you participate in this study again? | | |  | Yes or no |
|  | | If no, why? |  | Free response |
| **(13)** Would you recommend this study to a member of your family or a friend if they were given the chance to participate? | | |  | Yes or no |
| **(14)** Is there anything else you would like us to know? Please comment below. | | |  | Free response |
